# Device-measured baseline physical activity is associated with 24-h CGM glycemic responses and 12-week time-in-range after mixed exercise training in type 2 diabetes: a randomised crossover trial

**DOI:** 10.1186/s13102-026-01683-z

**Published:** 2026-04-27

**Authors:** Bin Gai, Min Yan, Guangxin Yang, Hao Shi, Yongliang Zhu, Xuewen Tian

**Affiliations:** 1 Graduate School of Education, Shandong Sport University, Jinan, 250102 China; 2 School of Exercise and Health, Tianjin University of Sport, Tianjin, China; 3 Institute of Sport Science, Shandong Sport University, Jinan, 250102 China

**Keywords:** Type 2 diabetes, Continuous glucose monitoring, Time-in-range, Mixed exercise training, Physical activity phenotype, Exercise adherence, Glycemic control

## Abstract

**Purpose:**

To evaluate whether baseline habitual physical activity (PA), modeled as continuous moderate-to-vigorous PA (MVPA), is associated with acute glycemic responses to different exercise modalities, and whether acute response is associated with 12-week glycemic status in older adults with type 2 diabetes.

**Methods:**

A total of 23 participants completed the acute crossover phase of the study, while the final longitudinal analysis for the 12-week intervention included 22 participants due to incomplete data from one individual. Continuous glucose monitoring quantified time in range (TIR, 3.9–10.0 mmol/L). Acute responses were analyzed using linear mixed models including modality, baseline MVPA, and modality × MVPA interaction terms, adjusted for age and sex. Associations between acute 24-h TIR after the mixed session and 12-week mean TIR were tested using Pearson correlation and HbA1c-adjusted partial correlation; stability was evaluated with leave-one-out cross-validation (LOOCV). Adherence interaction analyses were exploratory.

**Results:**

Evidence for effect modification by baseline MVPA in the acute phase was limited (Mixed × MVPA: β = -0.263, 95% CI -0.585 to 0.058; *P* = 0.109). Acute 24-h TIR after mixed exercise was associated with 12-week mean TIR (*r* = 0.887; partial *r* = 0.815; both *P* < 0.001), with stable LOOCV performance (R² = 0.729). Exploratory analyses suggested a baseline MVPA × adherence interaction for long-term TIR (β = 0.69; *P* = 0.031).

**Conclusion:**

Acute CGM response was strongly associated with longer-term glycemic status, whereas acute interaction evidence by baseline MVPA was not definitive. A short CGM-based exercise challenge may help characterize individual response patterns, although external validation is needed.

**Trial registration:**

Chinese Clinical Trial Registry, ChiCTR2400085258. Registered 04 June 2024. Retrospectively registered.

**Supplementary Information:**

The online version contains supplementary material available at 10.1186/s13102-026-01683-z.

## Introduction

Type 2 diabetes mellitus (T2DM) is a progressive metabolic disorder characterized by persistent hyperglycemia, necessitating sustained glycemic stability to mitigate cardiovascular and microvascular risks [1]. While structured exercise is a cornerstone of non-pharmacological management, current clinical guidelines increasingly advocate for mixed training—the integration of aerobic and resistance components—due to its superior efficacy in HbA1c reduction compared to single modalities [2, 3], particularly in individuals with elevated baseline HbA1c levels [4]. Exercise exerts dual glucoregulatory effects: a single bout can acutely lower 24-hour mean glucose and hyperglycemic excursions [5, 6], while chronic adherence to training promotes sustained improvements in insulin sensitivity, glycemic variability, and HbA1c [6].

However, a substantial proportion of individuals fail to derive expected benefits from standard exercise prescriptions, highlighting profound inter-individual heterogeneity [7]. This variability in response is well-documented across diverse populations and modalities [8] and is modulated by factors such as exercise dose, timing relative to meals, medication status, and baseline characteristics [4, 9, 10]. Yet, amidst these established determinants, a critical behavioral phenotype remains largely underexplored: baseline habitual physical activity (PA).

Sedentary behavior is strongly associated with poor glycemic control [11, 12], whereas higher levels of habitual physical activity correlate with lower HbA1c [13], reflecting a baseline state of favorable muscular adaptation [14]. While active individuals with T2DM can still derive glycemic benefits from additional structured exercise [15], sedentary patients—often characterized by poorer baseline metabolic health—may paradoxically exhibit a greater magnitude of improvement. The American Diabetes Association guidelines note that even minimal energy expenditure can enhance insulin action in sedentary adults [2], and those with greater baseline insulin resistance typically demonstrate the most significant sensitivity gains [11]. Consequently, sedentary individuals who adhere to long-term training often achieve superior relative reductions in HbA1c and increases in Time in Range (TIR) compared to their active counterparts matched for exercise dose. However, it remains unclear whether baseline physical activity phenotype specifically modulates the sensitivity to the mechanical load of mixed training. Without this knowledge, it remains difficult to identify which individuals are more likely to respond favorably to mixed training.

Furthermore, current exercise prescription often relies on repeated assessment over time, patients must train for months before efficacy can be assessed [16]. While the prognostic value of acute drug responses is established [17], it is unknown whether the acute glycemic response to a single mixed exercise session may help characterize longer-term adaptations. This gap persists despite growing recognition that individual responses to standard exercise doses are highly heterogeneous [18]. Additionally, the interplay between baseline phenotype and exercise adherence, specifically, whether high adherence is equally critical for everyone or if sedentary individuals can benefit even with suboptimal adherence, has not been elucidated.

Continuous glucose monitoring (CGM) offers a unique opportunity to capture these dynamic responses with high temporal resolution [19]. The metric TIR has emerged as a robust surrogate for long-term complications, yet its application in linking acute exercise sensitivity to chronic training outcomes is limited.

Therefore, the primary objective of this study was to determine whether the baseline physical activity phenotype modulates the acute glycemic efficacy of aerobic, resistance, and mixed exercise modalities in older adults with type 2 diabetes and to establish whether these acute responses can reliably predict long-term clinical outcomes. As a secondary objective, we explored how intervention adherence further influences these long-term metabolic adaptations. We hypothesized that baseline activity levels would significantly influence sensitivity to different exercise modalities and that individual acute responsiveness would remain associated with twelve-week glycemic control independent of baseline disease severity.

## Methods

### Study design

This study employed a two-phase design integrating a randomized, counterbalanced crossover trial (Phase 1) with a 12-week longitudinal intervention and follow-up (Phase 2). The study protocol and analytical framework were established and approved by the institutional review board before the start of data collection.

#### Phase 1 (Acute phase)

To evaluate acute exercise-induced glycemic excursions, all eligible participants completed three exercise modalities (Aerobic, Resistance, and Mixed) in a randomized, counterbalanced sequence. A minimum 48-hour washout period was mandated between sessions. Habitual physical activity was assessed via 7-day accelerometry prior to the intervention to characterize each participant’s baseline phenotype along a continuous activity spectrum. This allowed for the evaluation of baseline physical activity as a phenotypic moderator of modality-specific acute glycemic responses.

#### Phase 2 (Chronic phase)

Following the acute phase, participants underwent a 12-week supervised chronic intervention consisting of mixed exercise training. This longitudinal phase was designed to determine the prognostic value of individual acute responsiveness. Specifically, we evaluated whether the magnitude of the acute 24-hour post-exercise TIR (following the mixed modality) could serve as a reliable predictor of long-term glycemic adaptations under free-living conditions. All participants maintained their baseline oral hypoglycemic medication types and dosages throughout the 12-week intervention and no medication changes were reported during the study.

### Sample size determination

Sample size was calculated a priori based on the primary objective of establishing the predictive validity of acute glycemic responses for long-term outcomes. Anticipating a correlation coefficient of *r* ≥ 0.60, a minimum of 19 participants was required to achieve 80% power (α = 0.05, two-tailed; G*Power 3.1). The final sample of 22 participants provided > 85% power for this primary association. Additionally, for the acute crossover phase (Phase 1), the required sample size of 19 was determined to provide over 90% power to detect a medium-to-large effect size (Cohen’s f = 0.30) for within-subject differences across the three exercise modalities (Repeated Measures ANOVA, 3 measurements, α = 0.05).

Ultimately, 23 participants were enrolled, and the final analytical sample of *N* = 22 who completed all experimental phases exceeded the predetermined threshold required to maintain sufficient statistical power for both primary and secondary objectives.

### Study participants

#### Recruitment and ethics

Participant recruitment and data collection occurred between March 2023 and July 2023 through a combination of physician referrals at Qilu Hospital and community-based advertisements. Of 138 individuals screened, 108 were excluded for not meeting criteria such as stable medication or HbA1c levels, with detailed reasons provided in Supplementary Table S1. Consequently, 30 eligible participants were enrolled and randomly assigned to intervention sequences. In this study, older adults were defined as individuals aged 50 years and older. To ensure allocation concealment, an independent researcher prepared sequentially numbered, opaque, sealed envelopes (SNOSE), which were opened by the investigator only after informed consent was obtained. The study was registered with the Chinese Clinical Trial Registry (ChiCTR2400085258; Registered 04 June 2024; Retrospectively registered). Ethical approval was obtained from the Institutional Review Board of Shandong Center for Disease Control and Prevention (SDJK-2022-38-02) prior to the study commencement and conducted in accordance with the Declaration of Helsinki. All participants provided written informed consent prior to enrollment.

#### Blinding

Outcome assessors and data analysts were blinded to the intervention sequences until the completion of the study, though participants and exercise supervisors could not be blinded due to the nature of the exercise intervention.

### Participant flow and final sample

**Fig. 1:**
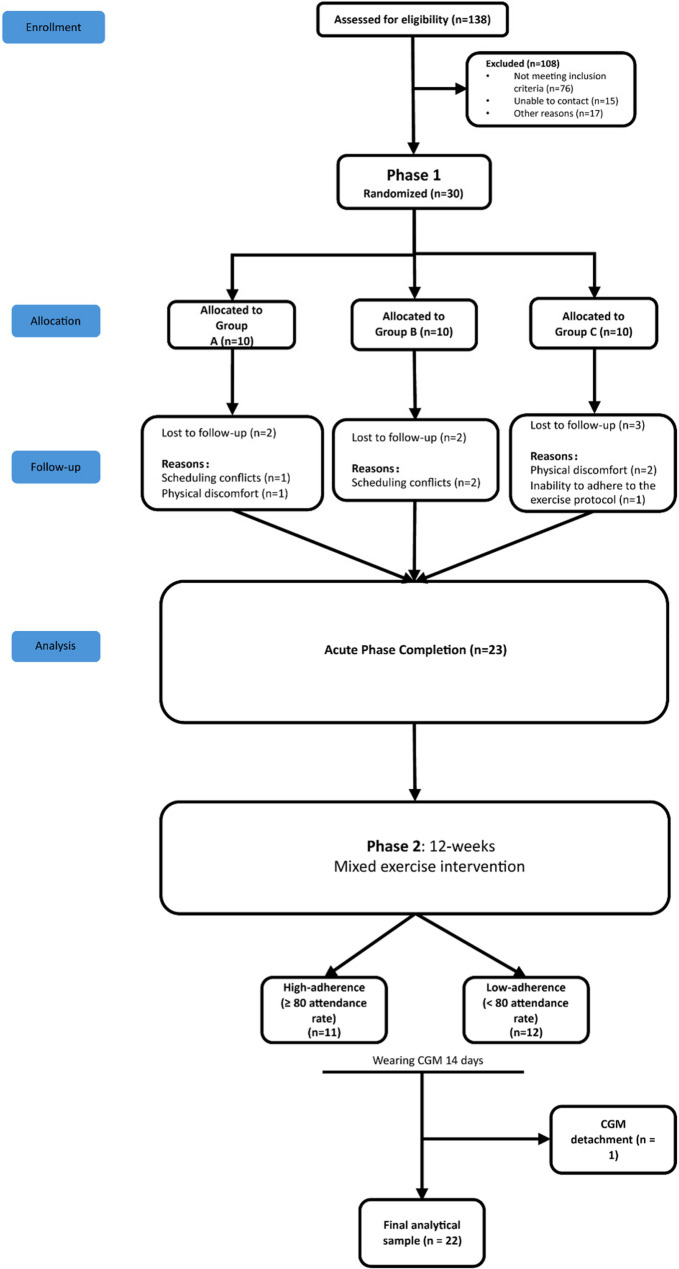
CONSORT flow diagram. An overview of participant progression through the trial stages, including extended interventions

Of the 30 participants initially randomized, 23 completed the Phase 1 acute crossover sessions (the 7 dropouts were due to personal schedule issues and physical discomfort). Baseline daily activity phenotyping was performed on these 23 individuals. For the Phase 2 chronic intervention analysis, 22 participants were included as one participant (P17) dropped out during the 12-week follow-up period due to personal reasons. The CONSORT flow diagram is presented in Fig. 1.

### Baseline physical activity assessment

Before the initiation of exercise interventions, all participants underwent a 7-day baseline physical activity assessment using triaxial accelerometers (ActiGraph wGT3X-BT; ActiGraph, Pensacola, FL, USA). Valid data were defined as at least 7 days of recording with ≥ 10 h of wear time per day. Daily MVPA was calculated using the Freedson cut-points. To maximize statistical power and preserve the full range of individual variability, baseline MVPA was treated as a continuous phenotypic variable in all primary interaction models. For visualization of acute response patterns in CGM profiles, the participants were secondary characterized by MVPA tertiles.

Following the completion of all baseline assessments, participants commenced the exercise intervention phase. Before the intervention, exercise safety and intensity were calibrated via comprehensive physiological testing. Cardiorespiratory fitness was assessed using a submaximal cycle ergometer protocol (20 W increments every 3 min) to determine peak heart rate for aerobic prescribing. Due to participants’ age, resistance intensity for elastic bands was individualized via handgrip strength and baseline tolerance rather than 1RM testing. Fasting venous blood was collected for baseline HbA1c and lipid profiling.

### Exercise intervention and dietary control

#### Phase 1

Participants completed three 40-minute duration-matched exercise sessions (Aerobic, Resistance, and Mixed) in randomized order, separated by ≥ 48-h washouts. Sessions were standardized to 09:45–10:45 and included 10-minute warm-ups/cool-downs. Aerobic consisted of continuous bodyweight circuits (60–75% peak HR). Resistance utilized individualized elastic band resistance (15–20 RM) targeting major muscle groups. Mixed combined 50% volume of both modalities. Detailed exercise protocols and movements are provided in Supplementary Methods. The supervised exercise intervention was conducted at Qilu Hospital. Adverse events, including hypoglycemia and excessive heart rate, were systematically monitored during all training sessions, with no serious adverse events reported. Following each session, participants consumed a standardized isocaloric meal (Supplementary Table S3).

#### Phase 2

Participants performed supervised mixed exercise sessions 3 times per week for 12 weeks. Adherence was calculated as the percentage of sessions attended, with participants stratified into High (≥ 80%) or Low (< 80%) adherence subgroups. This threshold is a widely recognized benchmark in medical research, particularly in pharmaceutical studies, for ensuring sufficient intervention exposure [20].

### CGM data processing and metrics

Interstitial glucose was monitored using a Flash Glucose Monitoring system (FreeStyle Libre, Abbott, USA). Throughout the entire 14-day Phase 1 intervention period, participants wore the CGM sensor continuously.​ This allowed for the capture of the three supervised exercise sessions. To ensure data quality, all sensors were calibrated according to manufacturer instructions.

#### Phase 1

To capture the specific metabolic sensitivity to each exercise modality while minimizing the confounding effects of immediate post-exercise physiological stress, the acute analysis window was strictly defined. Data were extracted for the 24-hour period commencing one hour post-exercise (specifically, from 10:45 on the exercise day to 10:45 on the following day). The primary outcome was Time in Range (TIR, 3.9–10.0 mmol/L), calculated as the percentage of time glucose values remained within this target range during the 24-hour window.

#### Phase 2

At the end of the 12-week intervention, participants underwent a second CGM assessment under free-living conditions without supervised exercise sessions. Sensors were worn for up to 14 days. To be included in the final analysis, participants were required to have at least 10 days (or > 70% data capture) of valid glucose readings. This threshold ensures that the derived metrics are representative of the participant’s chronic glycemic state [21]. For participants with 10–14 days of data, TIR was calculated based on their total valid monitoring duration. One participant was excluded from the chronic analysis due to sensor detachment resulting in insufficient data (< 3 days).

### Statistical analysis

Data are presented as mean ± SD unless otherwise specified. A per-protocol analysis was adopted, for the CGM datasets, sporadic missing values due to sensor signal loss were handled using mean imputation to maintain data integrity. Baseline characteristics were compared between high and low adherence groups using independent t-tests for continuous variables and Fisher’s exact tests for categorical variables to ensure cohort balance.

#### Phase 1

A Linear Mixed Model (LMM) was employed to evaluate the 24-h TIR responses following different exercise modalities. Exercise modality, baseline MVPA (as a continuous variable), and their interaction were included as fixed effects. Age and gender were incorporated as covariates to adjust for potential confounding. A random intercept for each subject was included to account for the repeated-measures crossover design.

#### Phase 2

The association between acute 24-h TIR (following mixed exercise) and 12-week mean TIR was evaluated using Pearson correlation coefficients. To ensure the robustness of this relationship independent of baseline disease severity, a partial correlation analysis was performed, adjusting for baseline HbA1c. The stability and predictive performance of this association were further validated using Leave-One-Out Cross-Validation (LOOCV), from which the cross-validated R-squared (R^2^) and Mean Absolute Error (MAE) were derived.

The moderating effect of intervention adherence on long-term outcomes was analyzed using an Ordinary Least Squares (OLS) regression model, testing the interaction between baseline MVPA and adherence group. Significant interactions were further explored via simple slope analysis to determine the relationship between habitual activity and chronic TIR within each adherence level.

Within-group changes in biochemical markers were assessed using paired t-tests. All statistical analyses were performed using Python v3.8, with two-tailed significance set at *P* < 0.05.

#### Patient and public involvement

Patients or the public were not involved in the design, conduct, or reporting of this study. The research questions and outcome measures were developed based on clinical guidelines and previous literature in exercise physiology for type 2 diabetes.

## Results

### Participant characteristics and baseline phenotyping

A total of 23 participants with T2DM (mean age 63.0 ± 9.6 years) were enrolled. The participants exhibited a wide spectrum of habitual physical activity, with baseline MVPA ranging from 1.6 to 89.6 min/day (mean: 35.8 ± 27.9 min/day). Baseline clinical markers reflected suboptimal metabolic control, including a mean HbA1c of 8.01 ± 2.12% and fasting glucose of 9.55 ± 3.21%.

For the 12-week longitudinal analysis, participants were characterized by their intervention adherence. Eleven participants demonstrated high adherence (attendance ≥ 80%), while 12 were classified into the low adherence group (< 80%). The mean adherence for the entire sample was 79.8 ± 12.5%, with a range of 58.3% to 100%. No significant differences were observed in baseline demographics, habitual activity levels, or biochemical profiles between the high and low adherence groups (all *P* > 0.05; Supplementary Table S2), ensuring a balanced baseline for longitudinal comparisons. One participant (P17) was excluded from the chronic analysis due to sensor failure, resulting in a final analytical sample of 22 for longitudinal outcomes.

### Acute glycemic responses post-exercise

The LMM analysis revealed a significant main effect for the mixed exercise modality compared to aerobic exercise at the baseline intercept (β = 15.56, 95% CI [1.09, 30.03], *P* = 0.035; Table 1). The regression coefficient for the interaction between mixed exercise and baseline MVPA was − 0.263 (95% CI [-0.585, 0.058], *P* = 0.109). For resistance exercise, the main effect was 11.35 (*P* = 0.124) and its interaction with baseline MVPA was − 0.132 (*P* = 0.421). Demographic variables, including age (β = 0.100, *P* = 0.856) and gender (β = 5.405, *P* = 0.600), were not significantly associated with acute TIR.

**Table 1:** Pairwise comparisons of 24-h TIR following acute exercise sessions

Variable	β	SE	z	*P* value	95% CI
Intercept	59.127	37.359	1.583	0.114	[-14.096, 132.349]
Modality: Mixed (vs. Aerobic)	15.562	7.382	2.108	0.035*	[1.093, 30.030]
Modality: Resistance (vs. Aerobic)	11.348	7.382	1.537	0.124	[-3.121, 25.816]
Gender: Male (vs. Female)	5.405	10.306	0.524	0.600	[-14.794, 25.604]
Baseline MVPA	-0.131	0.212	-0.618	0.537	[-0.547, 0.285]
Mixed × Baseline MVPA	-0.263	0.164	-1.605	0.109	[-0.585, 0.058]
Resistance × Baseline MVPA	-0.132	0.164	-0.805	0.421	[-0.454, 0.189]
Age	0.100	0.551	0.182	0.856	[-0.980, 1.180]
Group variance (random intercept)	521.492	15.420			

Linear mixed model estimates. Reference categories were Aerobic (modality) and Female (gender)

β Regression coefficient, *SE* Standard error, *CI* Confidence interval

* *P* < 0.05. z, Wald z-statistic

**Fig. 2:**
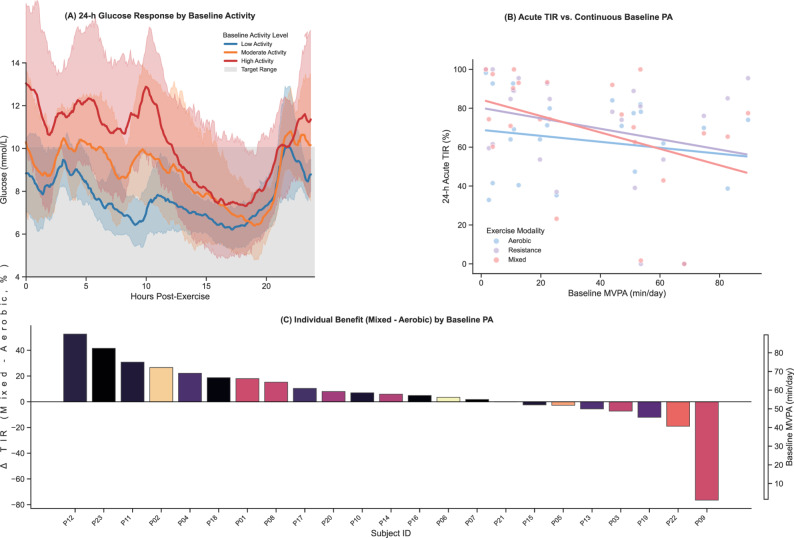
Acute glycemic responses to different exercise modalities stratified by baseline physical activity phenotype. **A** 24-hour continuous glucose monitoring (CGM) profiles. The excursion curves represent the mean glucose levels following aerobic, resistance, and mixed exercise sessions. Data are stratified by baseline MVPA levels for visualization, with the shaded area (3.9–10.0 mmol/L) representing the clinical target range. **B** Acute TIR response across the continuous spectrum of baseline MVPA. Linear regression slopes representing the relationship between habitual physical activity (Baseline MVPA) and 24-hour TIR for aerobic (blue), resistance (purple), and mixed (red) exercise modalities. **C** Individual therapeutic gain (ΔTIR) when switching from aerobic to mixed exercise

Visual analysis of the 24-hour glucose excursion curves (Fig. 2A) shows the glycemic profiles across baseline activity tertiles. The continuous relationship between baseline MVPA and acute TIR for each modality is displayed in Fig. 2B. Individual therapeutic gains (ΔTIR), calculated as the difference between mixed and aerobic exercise sessions, are presented in Fig. 2C. In the subset of participants with baseline MVPA below the group median, 75% (9/12) exhibited a positive ΔTIR, whereas the response distribution in individuals above the median was more variable.

### Acute responsiveness is associated with long-term glycemic outcomes

**Fig. 3:**
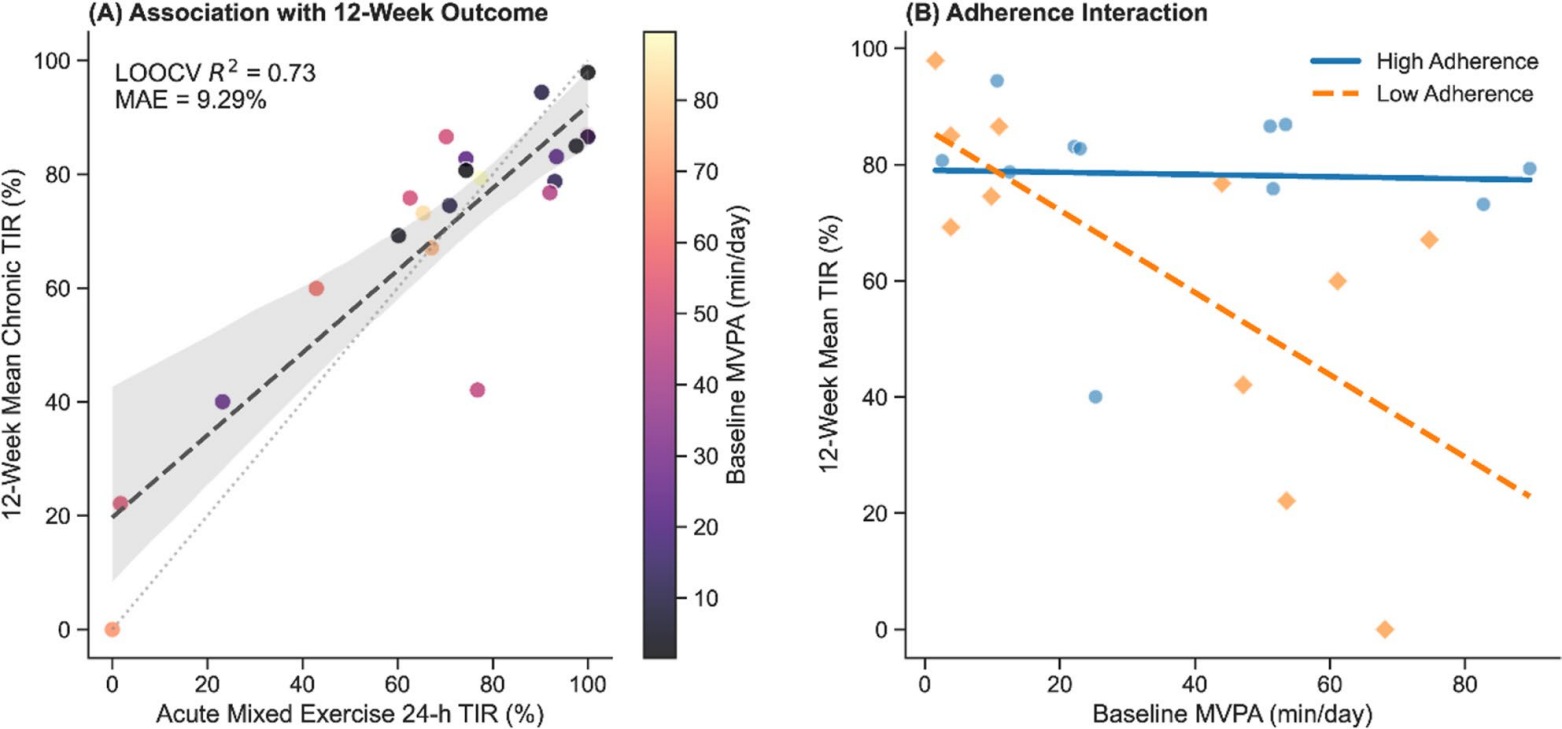
Acute glycemic responsiveness is associated with long-term glycemic control. Panel **A** shows the correlation between the acute 24 h post exercise TIR and the 12 week mean TIR. Panel **B** illustrates the moderating effect of intervention adherence on the relationship between baseline MVPA and 12 week TIR for the high adherence and low adherence groups

To evaluate the clinical relevance of acute glycemic challenges, we examined the association between the 24-hour TIR following a single session of mixed exercise and the mean TIR achieved over the subsequent 12-week intervention period (Fig. 3A).

Across the entire group (*n* = 22), a strong positive association was observed between acute and chronic TIR (Pearson *r* = 0.887, 95% CI [0.744, 0.952], *P* < 0.001), accounting for approximately 79% of the shared variance (R2 = 0.787). Importantly, this association remained robust after adjusting for baseline disease severity (HbA1c) using partial correlation analysis (Partial *r* = 0.815, 95% CI [0.592, 0.922], *P* < 0.001; Table 2). The LOOCV model demonstrated high predictive consistency (R2 = 0.729), with a Mean Absolute Error of only 9.29%.

**Table 2:** Pearson correlation coefficients between baseline physical activity phenotypes and acute TIR responses during Phase 1

Correlation Type		Value	95% CI (*r*)	*P*-value	Control Variable
Zero-order (Pearson)	r	0.887	[0.744, 0.952]	< 0.001	None
Partial Correlation	pr	0.815	[0.592, 0.922]	< 0.001	Baseline HbA1c
Robustness (LOOCV)	R^2^	0.729			
	MAE (%)	9.29			

*n* = 22. This table shows the correlation between baseline moderate-to-vigorous physical activity (MVPA) and the acute change in Time-in-Range

*CI* Confidence Interval, *MAE* Mean Absolute Error, *HbA1c* Glycated Hemoglobin, *LOOCV* Leave-One-Out Cross-Validation

To further explore the factors contributing to long-term success, we examined the moderating role of intervention adherence on the relationship between baseline physical activity and 12-week glycemic outcomes (Fig. 3B).

The OLS regression model revealed a significant interaction between baseline MVPA and adherence group (β = 0.69, 95% CI [0.07, 1.31], *P* = 0.031; Table 3). Simple slope analysis indicated that for participants with low adherence, higher baseline MVPA was paradoxically associated with lower chronic TIR (β = -0.71, *P* = 0.003). However, this negative trend was almost entirely mitigated in the high-adherence group (β_combined_ = -0.02, Fig. 3B).

**Table 3:** Moderation analysis of adherence on the relationship between baseline MVPA and 12-week TIR

Variable	β	SE	t	*P* value	95% CI
Intercept	86.320	9.257	9.324	< 0.001	[66.871, 105.769]
Adherence group: 1 (vs. 0)	-7.264	13.501	-0.538	0.597	[-35.629, 21.101]
Baseline MVPA	-0.708	0.211	-3.362	0.003	[-1.151, -0.266]
Baseline MVPA × Adherence group: 1	0.689	0.295	2.338	0.031*	[0.070, 1.309]

*n* = 22. The dependent variable is the TIR at 12 weeks. Adherence group 1 represents high adherence (≥ 80%) and group 0 represents low adherence (< 80%). Baseline MVPA is treated as a continuous variable. The term Baseline MVPA × Adherence group represents the interaction effect between habitual activity and intervention adherence

β Unstandardized regression coefficient, *SE* Standard error, *t* T-statistic, *CI* Confidence interval, *MVPA* Moderate-to-vigorous physical activity

*, *P* < 0.05

### Biochemical improvements

Over the 12-week intervention, significant improvements were observed in key clinical markers across all participants (Supplementary Table S4). HbA1c decreased by a mean of 1.08% (from 8.08 ± 2.14% to 7.00 ± 1.61%, *P* = 0.002), and fasting glucose reduced by 0.89 mmol/L (*P* = 0.044). Notably, the clinical benefit was adherence-dependent; the High Adherence group (*n* = 11) achieved a robust HbA1c reduction of 1.21% (*P* < 0.001), while the Low Adherence group (*n* = 11) showed a more modest improvement (0.95%, *P* = 0.038).

## Discussion

This study examined exercise responsiveness in older adults with type 2 diabetes and yields three findings. First, baseline habitual activity modulates the acute glycemic responses to varying exercise modalities, where individuals with lower initial activity levels tend to exhibit larger improvements following mixed exercise sessions. Second, the acute twenty-four-hour response to a single mixed exercise session is strongly associated with twelve-week glycemic outcomes, and this predictive relationship remains stable across the entire habitual activity spectrum, demonstrating high consistency regardless of an individual’s initial phenotype. Third, as a secondary exploratory finding, intervention adherence interacted with baseline activity to shape long-term control, indicating that while individuals with lower habitual activity show relatively preserved benefits, those with higher baseline activity may be more sensitive to reduced training frequency.

The superiority of mixed training for glycemic management in type 2 diabetes has been supported by prior trials and consensus recommendations [2, 3, 22]. However, relatively few studies have examined habitual physical activity as a continuous moderator when comparing modality-specific glycemic responses. In the present study, participants with higher habitual activity levels showed minimal modality-specific differences in acute TIR, whereas the advantage of mixed training was more prominent at the lower end of the activity spectrum, with resistance exercise showing a similar directional pattern. This is consistent with evidence that individuals with low habitual activity can exhibit meaningful improvements in insulin action with relatively small increases in energy expenditure [2, 11]. Notably, in our data, this enhanced responsiveness was primarily observed after sessions that included a resistance component, suggesting that resistance-related stimuli may be a key driver. Mechanistically, skeletal muscle contractions during resistance exercise promote GLUT4 translocation and increase insulin-independent glucose uptake [23–25]. In older adults with chronically low activity, lower muscle mass and functional reserve may further amplify metabolic responsiveness to resistance-based stimuli.

International guidelines for type two diabetes recommend at least one hundred and fifty minutes of moderate-to-vigorous physical activity per week to optimize metabolic control [26]. While our participants averaged approximately thirty-six minutes per day, the significant individual variability underscores that habitual activity is a continuous spectrum rather than a binary threshold. This perspective aligns with recent consensus statements emphasizing both the reduction of sedentary time and the implementation of structured exercise [11, 12, 27]. By capturing the transition from sedentary behavior to an active lifestyle, our findings suggest that individuals at the lower end of this spectrum exhibit unique sensitivity to potent stimuli like resistance-based mixed exercise. Such a continuum-based approach provides a more nuanced understanding of exercise responsiveness and supports the development of personalized exercise prescriptions in diabetes care.

The strong association between the 24-hour response to a mixed exercise session and the 12-week outcomes suggests that a standardized, short-term continuous glucose monitoring challenge may provide significant prognostic value for long-term clinical success. Conceptually, this aligns with broader physiological frameworks that utilize early markers to tailor medical interventions, although such methods require careful validation before broad clinical deployment [16, 17]. From a clinical perspective, this acute response may have potential relevance as a standardized exercise challenge, although further validation is needed before clinical use. It may help inform future efforts to identify inter-individual differences in response to mixed exercise training. The stability of this association, further supported by LOOCV analysis, warrants further evaluation of acute responsiveness as a potential indicator. While interpreting individual differences requires caution due to biological variability and potential measurement error in modest samples [18, 28], the consistency of this correlation across the activity spectrum underscores its potential for personalized diabetes management.

While the strong correlation confirms our primary hypothesis, a portion of the long-term variance remained unexplained. Our exploratory analyses suggest that intervention adherence serves as a key factor in this residual variability, with its impact being moderated by baseline activity levels. Individuals who are already relatively active may require consistent exposure to structured and adequately dosed training to maintain or further extend glycemic benefits. This pattern is conceptually consistent with dose–response principles often discussed in resistance training adaptations [29]. In contrast, individuals with lower baseline activity may derive larger per-session benefits, reflecting greater marginal gains with initial training exposure [2, 11]. These results provide clear cues for personalized clinical support, highly active patients may require strategies focused on strict exercise adherence to sustain their glycemic status, while less active individuals should first prioritize the consistent initiation of exercise and the reduction of sedentary time before scaling the training dose.

Alternative explanations should also be considered, as high adherence is often a surrogate for broader self-management competence. Participants with superior attendance may also exhibit better dietary control, regular medication habits, and consistent sleep hygiene, each of which independently influences glucose stability [30, 31]. Therefore, the observed interactions should be interpreted as a signal for personalized clinical guidance rather than a simple causal conclusion.

Several limitations warrant consideration. While the randomized crossover design provided strong internal control for acute comparisons, the modest sample size may limit the precision of subgroup interaction estimates. Additionally, adherence was measured via session attendance, which may not fully reflect the actual physiological load or unsupervised activity. Finally, although our analysis of habitual activity as a continuous spectrum supported the primary findings, larger independent studies are needed to confirm the stability of these predictive models and interaction effects. In addition, the adherence interaction analyses were exploratory and should be interpreted cautiously. External validation in larger independent samples is needed before broader clinical interpretation. Future research should incorporate multidimensional adherence metrics, such as training intensity and external load, to better define the dose-response relationship across diverse physical activity phenotypes.

## Conclusion

Baseline physical activity levels are associated with both the acute and long-term glycemic responses to exercise in older adults with type two diabetes. Individuals at the lower end of the activity spectrum tend to exhibit more pronounced acute improvements following mixed and resistance exercise, whereas long-term outcomes in those who are more active appear more closely linked to intervention adherence. Notably, the acute 24-hour response to a single mixed exercise session is strongly associated with 12-week clinical outcomes, suggesting its potential value as an informative marker for individual adaptations. These findings highlight the importance of considering baseline phenotypes when designing exercise interventions and may help inform future efforts toward more individualized exercise support in diabetes care.

## Supplementary Information

Supplementary Material 1.

Supplementary Material 2.

## Data Availability

All de-identified data and analysis code used in this study will be made publicly available in a Zenodo repository upon publication. The repository will include participant-level summary data sufficient to reproduce the main results and all Python scripts used to generate the tables and figures presented in the manuscript. https://zenodo.org/records/18599292?preview=1&token=eyJhbGciOiJIUzUxMiJ9.eyJpZCI6ImNhYjlmYjZlLWU1MzQtNGMwYy1hYjM0LTlmMjM2YjVjODM5MCIsImRhdGEiOnt9LCJyYW5kb20iOiJiYzcyMTIxOTdlNDkxMWJmNDE4ZDYyN2NhODgyNjI4YyJ9.udScz6R2c_3tDr_PDmk22zjoaZq-YK5he0wKfQZmbMntpoAX8qdpxb52JdM4djzvjMqUj2ridgsWEFSMVFCfMQ.
