# Supplementary material for: Device-measured baseline physical activity is associated with 24-h CGM glycemic responses and 12-week time-in-range after mixed exercise training in type 2 diabetes: a randomised crossover trial
Source: BMC Sports Sci Med Rehabil. 2026 Apr 27;18:272. doi: 10.1186/s13102-026-01683-z (PMC13261961; doi:10.1186/s13102-026-01683-z)
Supplement: Supplementary file 2 — Supplementary Material 2. [file 13102_2026_1683_MOESM2_ESM.docx]

**Supplementary Methods**

**Participant Recruitment and Screening Details**

A total of 138 individuals expressed initial interest in the study through community advertisements and hospital referrals. Following a structured screening process, 108 individuals were excluded. The specific inclusion and exclusion criteria are summarized in Supplementary Table S1 below

Table S1 Inclusion and Exclusion Criteria for Study Participation

| Category | Criteria |
| --- | --- |
| Inclusion Criteria | - Aged 45–70 years  - Diagnosed with Type 2 Diabetes Mellitus (T2DM) confirmed by 75g OGTT (ADA criteria)  - Unstable fasting glucose (≥ 7.0 mmol/L on ≥ 3 mornings within 2 weeks)  - Receiving stable oral hypoglycemic agents (primarily metformin) for ≥ 3 months  - Capable of using an NFC-enabled smartphone for CGM data upload  - Able to participate in moderate-intensity physical activity (as confirmed by cardiopulmonary exercise testing)- Provided written informed consent |
| Exclusion Criteria | - Current insulin use- History of cardiovascular disease or severe diabetic complications  - Systolic BP ≥ 160 mmHg or diastolic BP ≥ 100 mmHg  - Any contraindication to moderate-intensity exercise  - Cognitive impairment, physical disability, or other conditions limiting exercise participation  - Participation in structured physical training within the past 6 months- Declined or withdrew consent during screening |

**CGM processing and outcome definitions**

TIR was defined as the percentage of CGM readings between 3.9 and 10.0 mmol/L. CGM values that were non-physiological (≤0 mmol/L) were treated as invalid and removed prior to analysis.

Acute post-exercise outcomes were derived from a prespecified 24-h window anchored to the acute mixed-exercise session end time. Specifically, the acute 24-h period commenced at 10:45 and extended to 10:45 the following day. For each participant and modality, acute 24-h TIR was calculated as the proportion of valid CGM samples within the target range during this window. To ensure adequate data quality for acute endpoints, acute 24-h metrics were computed only when a minimum number of valid CGM samples were available within the window (threshold: ≥201 samples).

Long-term free-living outcomes were computed from the 12-week CGM dataset using a pooled approach. For each participant, 12-week mean free-living TIR (“pooled TIR”) was calculated by pooling all valid CGM readings across the monitoring period and computing the percentage of readings within 3.9–10.0 mmol/L. As a robustness check, overall CGM capture was quantified for each participant as the proportion of non-missing CGM samples relative to the expected total number of samples given the device sampling interval and monitoring duration; sensitivity analyses were repeated in the subset meeting an overall capture threshold of ≥70%.

**Detailed Exercise Protocols**

Phase 1: Acute Exercise Modalities

All sessions (AE, RE, ME) were duration-matched (40 minutes of main activity) and performed between 09:45 and 10:45 to control for circadian rhythms. All exercise sessions were supervised by certified exercise specialists or graduate students in sports science

Aerobic Exercise (AE) Protocol: The AE session utilized a bodyweight-based continuous circuit. Participants performed 8 cycles of the following 5 movements, with each movement lasting 60 seconds and no rest between exercises: Standing high knees, Lateral side steps with arm swings, Standing oblique crunches (elbow-to-knee), Modified jumping jacks (low impact), Stationary marching with exaggerated arm movements

Intensity was monitored to maintain a HR zone of 60–75% of the participant's peak HR determined via baseline ergometer testing.

Resistance Exercise (RE) Protocol: The RE session utilized individualized elastic bands (TheraBand) targeting six major muscle groups. Intensity was set to 15–20 repetition maximum (RM), defined as the level of resistance eliciting volitional fatigue within that range. Participants performed 4 sets of the following exercises with 1-minute inter-set rest intervals: Bicep curls, Triceps extensions, Lateral raises, Squats, Bent-over rows, Chest flys.

Mixed Exercise (ME) Protocol: The ME modality integrated both types to ensure volume consistency:

- First 20 minutes: 2 sets of each exercise from the RE protocol (50% of standalone RE volume).
- Final 20 minutes: 4 cycles of the AE protocol (50% of standalone AE volume).

2. Phase 2: 12-Week Chronic Intervention

Participants attended supervised ME sessions three times per week. Training intensity was re-evaluated every 4 weeks via RPE (Borg scale) and band tension adjustments to ensure progressive overload while maintaining the 15–20 RM and 60–75% HR intensity targets.

**Dietary Standardization**

To mitigate the confounding effects of postprandial glycemic excursions, a standardized meal protocol was implemented during the acute phase. Following each exercise session, all participants consumed an identical, isocaloric lunch at 11:30 AM. The meal provided approximately 560 kcal with a balanced macronutrient distribution (45–50% carbohydrate, 20–25% protein, and 25–30% fat). Meals were prepared by hospital nutrition staff to ensure consistency across all intervention days. Participants were supervised to ensure 100% consumption within a 20-minute window. Water was permitted ad libitum, but no additional caloric intake was allowed during the subsequent post-exercise monitoring period. The detailed composition of the standardized meal is provided in Supplementary file Table S2.

**Statistical analyses reported in Supplementary table**

**Supplementary Tables**

Table S2.Baseline characteristics of the study participants stratified by subsequent intervention adherence.

| Variable | Total Cohort (N=23) | High Adherence (n=11) | Low Adherence (n=12) | P-value |
| --- | --- | --- | --- | --- |
| Demographics |  |  |  |  |
| Age (years) | 63.0 ± 9.6 | 62.1 ± 10.2 | 63.8 ± 9.3 | 0.68 |
| Gender (M/F) | 13M / 10F | 6M / 5F | 7M / 5F | 0.92 |
| Weight (kg) | 71.7 ± 12.0 | 70.2 ± 11.5 | 73.1 ± 12.8 | 0.58 |
| Physical Activity |  |  |  |  |
| MVPA (min/day) | 35.8 ± 27.9 | 37.2 ± 29.5 | 34.5 ± 27.8 | 0.82 |
| Sedentary Time (min) | 1042 ± 134 | 1035 ± 128 | 1049 ± 145 | 0.81 |
| Clinical Markers |  |  |  |  |
| HbA1c (%) | 8.01 ± 2.12 | 7.95 ± 1.98 | 8.06 ± 2.31 | 0.90 |
| Glucose (mmol/L) | 9.55 ± 3.21 | 9.38 ± 3.05 | 9.71 ± 3.52 | 0.81 |
| Total Cholesterol (mmol/L) | 5.22 ± 1.15 | 5.15 ± 1.10 | 5.28 ± 1.25 | 0.79 |
| Triglycerides (mmol/L) | 1.84 ± 1.27 | 1.75 ± 0.95 | 1.92 ± 1.51 | 0.75 |
| HDL-C (mmol/L) | 1.30 ± 0.26 | 1.32 ± 0.24 | 1.28 ± 0.28 | 0.72 |

Table S3. Standardized lunch composition provided after each exercise session

| **Food Item** | **Amount** | **Function / Nutritional Role** |
| --- | --- | --- |
| Brown rice | 150 g | Complex carbohydrates; low glycemic index (primary CHO) |
| Steamed chicken breast | 100 g | Lean protein; supports post-exercise recovery |
| Stir-fried broccoli and carrots | 150 g | Dietary fiber, antioxidants, vitamins |
| Soybean sprouts with 1 egg | 80 g | Mixed protein and fiber; improves satiety |
| Seaweed soup | 200 ml | Hydration; low calorie; iodine and trace minerals |
| Water | Ad libitum | No additional glycemic load |

Table S4. Changes in biochemical parameters following the 12-week mixed exercise intervention.

| Variables | Group | Baseline | Post-12 Weeks | Change (Δ) | P-value |
| --- | --- | --- | --- | --- | --- |
| Glycemic Control |  |  |  |  |  |
| HbA1c (%) | Total | 8.08 ± 2.14 | 7.00 ± 1.61 | -1.08 | 0.002 |
|  | High | 7.82 ± 1.54 | 6.61 ± 0.98 | -1.21 | <0.001 |
|  | Low | 8.35 ± 2.65 | 7.40 ± 2.01 | -0.95 | 0.038 |
| Fasting Glucose (mmol/L) | Total | 9.59 ± 3.28 | 8.70 ± 2.50 | -0.89 | 0.044 |
|  | High | 9.12 ± 2.80 | 7.97 ± 1.85 | -1.15 | 0.012 |
|  | Lo | 10.06 ± 3.78 | 9.43 ± 2.92 | -0.63 | 0.156 |
| Lipid Profile |  |  |  |  |  |
| Total Cholesterol (mmol/L) | Total | 4.88 ± 0.95 | 4.39 ± 0.82 | -0.49 | <0.001 |
|  | High | 4.75 ± 1.02 | 4.18 ± 0.75 | -0.57 | 0.004 |
|  | Low | 5.01 ± 0.89 | 4.60 ± 0.88 | -0.41 | 0.021 |
| Triglycerides (mmol/L) | Total | 1.92 ± 1.15 | 1.65 ± 0.92 | -0.27 | 0.082 |
|  | High | 1.85 ± 0.98 | 1.48 ± 0.72 | -0.37 | 0.054 |
|  | Low | 1.99 ± 1.34 | 1.82 ± 1.09 | -0.17 | 0.412 |
| HDL-C (mmol/L) | Total | 1.12 ± 0.28 | 1.18 ± 0.25 | +0.06 | 0.115 |
|  | High | 1.15 ± 0.31 | 1.24 ± 0.28 | +0.09 | 0.068 |
|  | Low | 1.09 ± 0.25 | 1.12 ± 0.22 | +0.03 | 0.485 |

Note: Data are Mean ± SD. The total cohort (N=22) was stratified into the High Adherence group (n=11) and the Low Adherence group (n=11) based on their completion of the prescribed exercise volume. P-values were determined via paired-sample t-tests. Bold values denote statistical significance (P < 0.05)
